# Addressing Acute Stress among Professionals Caring for COVID-19 Patients: Lessons Learned during the First Outbreak in Spain (March–April 2020)

**DOI:** 10.3390/ijerph182212010

**Published:** 2021-11-16

**Authors:** José Joaquín Mira, Ángel Cobos-Vargas, Maria Pilar Astier-Peña, Pastora Pérez-Pérez, Irene Carrillo, Mercedes Guilabert, Virtudes Pérez-Jover, Cesar Fernández-Peris, María Asunción Vicente-Ripoll, Carmen Silvestre-Busto, Susana Lorenzo-Martínez, Jimmy Martin-Delgado, Carlos Aibar, Jesús Aranaz

**Affiliations:** 1 Alicante-Sant Joan Health Department, 03013 Alacant, Spain; 2 Health Psychology Department, Miguel Hernandez University, 03202 Elche, Spain; icarrillo@umh.es (I.C.); mguilabert@umh.es (M.G.); v.perez@umh.es (V.P.-J.); c.fernandez@umh.es (C.F.-P.); suni@umh.es (M.A.V.-R.); 3 Quality and Patient Management, San Cecilio Clinical University Hospital, 18016 Granada, Spain; angel.cobos.sspa@juntadeandalucia.es; 4 “La Jota” Health Centre, 50015 Zaragoza, Spain; mpastier@gmail.com; 5 Spanish Society for Quality Care, 33003 Oviedo, Spain; pasperper@gmail.com; 6 Osasunbidea, 31002 Pamplona, Spain; msilvesb@yahoo.es; 7 Quality and Patient Management Department, Alcorcon Foundation University Hospital, 28922 Alcorcon, Spain; slorenzom@salud.madrid.org; 8 Atenea Research Group, Foundation for the Promotion of Health and Biomedical Research, 03550 Sant Joan D’ Alacant, Spain; jimmy.martind@umh.es; 9 Preventive Medicine Department, Lozano Blesa Clinical University Hospital, 50009 Zaragoza, Spain; caibar@posta.unizar.es; 10 Preventive Medicine Department, Ramón y Cajal University Hospital, 28034 Madrid, Spain; jesusmaria.aranaz@salud.madrid.org

**Keywords:** SARS Virus, pandemic, acute stress, fatigue, moral damage, moral labour, burnout, well-being, health personnel, hospitals, primary health care

## Abstract

*Objectives*: To describe lessons learned during the first COVID-19 outbreak in developing urgent interventions to strengthen healthcare workers’ capacity to cope with acute stress caused by health care pressure, concern about becoming infected, despair of witnessing patients’ suffering, and critical decision-making requirements of the SARS-CoV-2 pandemic during the first outbreak in Spain. *Methods*: A task force integrated by healthcare professionals and academics was activated following the first observations of acute stress reactions starting to compromise the professionals’ capacity for caring COVID-19 patients. Literature review and qualitative approach (consensus techniques) were applied. The target population included health professionals in primary care, hospitals, emergencies, and nursing homes. Interventions designed for addressing acute stress were agreed and disseminated. *Findings*: There are similarities in stressors to previous outbreaks, and the solutions devised then may work now. A set of issues, interventions to cope with, and their levels of evidence were defined. Issues and interventions were classified as: adequate communication initiative to strengthen work morale (avoiding information blackouts, uniformity of criteria, access to updated information, mentoring new professionals); resilience and recovery from physical and mental fatigue (briefings, protecting the family, regulated recovery time during the day, psychological first aid, humanizing care); reinforce leadership of intermediate commands (informative leadership, transparency, realism, and positive messages, the current state of emergency has not allowed for an empirical analysis of the effectiveness of proposed interventions. Sharing information to gauge expectations, listening to what professionals need, feeling protected from threats, organizational flexibility, encouraging teamwork, and leadership that promotes psychological safety have led to more positive responses. Attention to the needs of individuals must be combined with caring for the teams responsible for patient care. *Conclusions*: Although the COVID-19 pandemic has a more devastating effect than other recent outbreaks, there are common stressors and lessons learned in all of them that we must draw on to increase our capacity to respond to future healthcare crises.

## 1. Introduction

The rapid spread of the COVID-19 pandemic and the adverse conditions in which health care was initially carried out affected healthcare institutions’ teams’ wellbeing and work morale. They have led to acute stress reactions among health professionals, specifically increased intensity during that first outbreak [1,2,3]. This situation also threatened their ability to care for patients and offer an adequate quality of care to which professionals and patients were used to.

The overload caused by the rapid increase of cases, the uncertainty about their clinical outcome, and the emotional impact of seeing a significant number of patients dying (many of them alone), added to the constant changes in instructions, the break in the supply chain, or the fear of catching (or taking home the SARS-CoV-2) impacted the emotional balance of professionals. This impact is now becoming more evident as new data emerges [4,5,6].

Although healthcare professionals are used to acute stress derived from attending their duties during complex procedures worldwide, their physical and mental overload and effort have been very uneven [7]. There has been an urgent need to intervene to preserve the emotional stability of health professionals [8], indispensable to face the challenge of the new coronavirus.

The experience of previous outbreaks, together with intervention efforts being communicated, initially through social networks and other informal channels, and subsequently in scientific forums, led to proposals that sought to mitigate the psychological impact COVID-19 patient care had on institutions, staff and healthcare workers [9,10]. The approach of these interventions has been similar [6,11,12,13], and their implementation was motivated by the urgent need to act so that the entire healthcare workforce felt supported. Three strategic principles were pointed out [14]: providing leadership focused on resilience, structuring crisis communications to provide information and empowerment, and creating a continuum of staff support within the organization.

In the first wave, Spain was one of the countries where the incidence of COVID-19, mortality and the number of healthcare providers infected were higher. The alarming growth of discouraged professionals compromised the care of COVID-19 patients, fueling the creation of our task force. It was activated on 11 March 2020, following the first observations of acute stress reactions among healthcare professionals in Spanish hospitals and primary care centers. On that date, the number of confirmed patients with COVID-19 was 9801, and there had been 196 deaths. Five days later, a total of 47,800 cases and 1532 deaths had been recorded, exceeding the capacity of a growing number of professionals to cope with stress. During the first outbreak near a quarter of COVID-19 patients were healthcare workers [15]. Spain sadly topped the ranking of infected professionals [16]. This added to the overload and uncertainty caused added concern and distress among this group.

In Spain and different countries and institutions worldwide, interventions had to be implemented quickly to address this challenge and ensure that professionals found sufficient support to care for patients. These interventions were designed based on interventions in other health crises, in emergencies, and by combining different strategies adapted to the changing needs of the moment. The urgency of the moment made it necessary to take decisions and propose alternatives that were not known to be effective in this pandemic but had proved useful on previous occasions. Early reports were that professionals were becoming discouraged and that compassion fatigue, moral conflict, fear, material shortages and acute stress reactions were beginning to affect their ability to care for COVID-19 and other patients [17]. The time was ripe for proposals based on the needs that the centers themselves were identifying.

The main objective of this study is to describe the lessons learned during the first COVID-19 outbreak in the development of urgent interventions to strengthen healthcare workers’ capacity to cope with acute stress caused by health care pressure, critical decision-making requirements, concern about becoming infected, despair of witnessing patients’ suffering and the perception of seeing limited their ability to perform the task properly during the first outbreak of the SARS-CoV-2 pandemic in Spain. It includes a description of our approach and experience to share and reflections to better prepare for future crises. 

## 2. Materials and Methods

This intervention had a two-step approach. Firstly, recurring issues were analyzed. Secondly, appropriate interventions were identified to address each of them. This study combined a pragmatic literature review to identify and select impacts due to previous outbreaks and the implemented interventions to face. Moreover, a qualitative approach to collect data from several healthcare institutions from all over the country to identify the impact dues to caring for COVID-19 patients, undermining work morale and responsible for the acute stress experienced by healthcare personnel. Finally, the working group applied the consensus conference technique to agree upon brief interventions to address acute stress considering the level of evidence of the recommendations, following SIGN guidelines [18]. They should focus on the most common identified problems and, as far as possible, strengthen the work morale of the individuals and work teams. The identification and development phase of the intervention proposal was carried out expressly due to the urgency of the situation between 11 and 25 March 2020. The study protocol was approved by the Research Ethics Committee of the Hospital de Sant Joan (Alicante, Spain).

### 2.1. Scope and Objective of the Intervention 

To provide resources to strengthen the capacity of hospital and primary care healthcare professionals to restore work morale and cope with the acute stress experienced during the first wave of the COVID-19 pandemic. These interventions should be accepted and easily accessible for healthcare professionals. They should also be simple, self-applied, not time-consuming, and reach as many professionals as possible. Given the urgency, diversity of situations and personal experiences, these resources were designed to be available and used according to the needs of each moment and professional.

### 2.2. Population’s Target. Spanish Healthcare Workforce 

#### 2.2.1. Identification of Crucial Groups Involved 

Studies have identified higher acute stress among healthcare professionals of specific departments caring directly for COVID-19 patients [17]. The most vulnerable groups were healthcare personnel from Emergency and Critical Care Services, Home Hospitalization, Critical Care and Resuscitation, Internal Medicine, Pneumology and Infectious Diseases, and primary care. The healthcare professionals in training were particularly affected due to their transfer to assume the treatment of COVID-19 patients regardless of their background. 

#### 2.2.2. Core Group Constitution

At the beginning of March 2020, the Spanish research group on second victims was invited by several hospitals to propose interventions to give emotional support to healthcare professionals in whom symptoms of distress are already observed. This group had experiences in supporting interventions for professionals who had been involved in a safety incident with severe consequences for patients [19], made up of physicians, psychologists, nurses and pharmacists from both settings hospitals and primary care. This group expanded with professionals from several centers’ occupational health, mental health, and preventive medicine services. The Core Group that led this intervention included 28 professionals with clinical, management and academic profiles from 8 of the 17 Spanish autonomous communities. All of them were involved in the organization of the responses to the challenge caused by the outbreak in their healthcare institutions.

#### 2.2.3. Perspectives and Preferences of the Target Population

Barriers and strengths that would facilitate the dissemination of the approach and consensus recommendations for strengthening work morale and coping with acute stress were analyzed.

#### 2.2.4. Previous Experiences

An express review of published studies on the impact on healthcare professionals of previous pandemics, care of patients in terrorist attacks and natural disasters, and interventions to cope with these situations was conducted.

#### 2.2.5. Factors Responsible for Acute Stress during the First Outbreak

In parallel, a consensus was reached on common problems during the COVID-19 pandemic in hospitals or primary care. Also, to identify proposals to minimize this impact on healthcare professionals.

### 2.3. Literature Review

#### 2.3.1. Method of Documentation and Search for Evidence

The following databases were searched: ProQuest, Science Direct, Scopus, and MEDLINE. MeSH terms mental health, wellbeing, healthcare personnel, outbreak, and COVID-19 were combined by the Boolean operator AND (restricted terms to title and abstract) to localize references published in English and Spanish until March 2020. Google and Google Scholar and preprint archives were searched using key phrases in line with the search strategy. Lastly, grey literature and hand searching was employed as sources of information. Eligibility criteria included research conducted within the COVID-19 outbreak context, describing the impact. Exclusion criteria included studies on the general population’s impact on patients or groups other than health professionals.

#### 2.3.2. Data Extraction

VPJ, IC and MG read the titles and eliminated duplicates. After reading abstracts, relevant studies were retained, and the rest were discarded. A fourth researcher (JJM) resolved disagreements to assess the relevance of the studies. A description was made of the problems identified and the interventions to address them in each study. The following categories were established to extract information from each of the studies considered relevant for this study: the challenge to be addressed, country, year, target population, sample size, type of intervention, results. Mendeley and Excel were used to manage the references analyzed and share the information among the team members.

#### 2.3.3. Quality Evaluation of Included Articles

The Appraisal tool for Cross-Sectional Studies (AXIS tool) was used to assess published studies about the impact of the previous and current outbreak [20]. AXIS comprises 20 questions to assess mainly on the presented methods and results. It focuses on whether the published findings of a study are credible and reliable should relate to the aims, methods, and analysis of what is reported and not on the interpretation. AXIS was applied to the identified articles.

#### 2.3.4. Data Synthesis

The narrative synthesis approach was used. Key findings responding to essential questions defined previously by the research team were considered describing current understanding.

### 2.4. Field Study

#### 2.4.1. Participants

The 28 members of the Core Group (10 hospital and primary care area managers, four psychiatrists, three occupational health physicians, four nurses, four psychologists, one pharmacist, two others).

#### 2.4.2. Setting

Thirteen healthcare institutions from eight autonomous communities in Spain.

#### 2.4.3. Identifying the Impact of the Outbreak

Using the previous literature review analysis, categories were identified and generated to prepare structured questions to understand better and collect data in Spanish healthcare institutions. The most common problems were identified in terms of inability to cope with the situation, damage to work morale, breakdown of work teams, and psychological disturbance. Subsequently, a qualitative approach was used to capture professionals’ experiences in 12 primary care centers and 13 hospitals. Each of the Core Group members led their respective teams and were responsible for collecting the most relevant problem situations and sharing with the rest of the partners. Structure content analysis was used to code the information provided by the core group members regarding the impact of the outbreak. JJM, MG and IC made integration and coding of verbatims in a dataset to share this information among the members of the Core Group, who contributed to identifying the most common problems and their origins. All the nuances and experiences were consolidated in a table using Excel. This dataset was shared using e-mail and WhatsApp applications. Ideas were grouped as the information became saturated (spontaneity and consistency among the different informants). Based on the classification of the information in the categories of analysis, a second round was made incorporating possible interventions carried out from the experience of the core group members to respond to the problem situations raised. 

#### 2.4.4. Development of Support Interventions

First, for each coded problem in categories, the core group, guided mainly by mental and occupational health experts’, suggested specific interventions. Second, these interventions linked to identified problems were adapted to the current context demands. Barriers that could prevent or facilitate the implementation of these interventions and the expected benefits were also analyzed. Third, each core group member received all the information and assessed the adequacy of the proposed interventions, their feasibility and acceptability by the healthcare workers in their respective institutions. Also, produced feedback to improve the interventions considering the proposals collected. Fourth, the selection of interventions considered the available evidence and the successive feedback produced by healthcare workers in each institution participating in this study to improve these interventions before dissemination. Professionals with expertise in the development of audiovisual materials collaborated in this task. After successive reviews, the agreement among members of the core group determined the recommended resource proposals to address the demands caused by the pandemic.

#### 2.4.5. Criteria for Evidence Quality

Lastly, the interventions were classified according to the study design. SIGN [18] was used to determine the quality of the evidence and the strength of the recommendation [21]. SIGN uses a numeric and symbol scale to provide levels of evidence. The scale can range from 1++ (High-quality meta-analysis), 1+ (Well-conducted meta-analysis, systematic reviews), 1− (meta-analysis and systematic reviews), 2++ (High-quality systematic reviews of case-control or cohort studies), 2+ (well-conducted case-control or cohort studies), 2− (case-control or cohort studies with high risk of bias), 3 (non-analytic studies) and 4 (Expert opinion). No article was excluded based on this information, although it was taken into account when prioritizing recommendations.

#### 2.4.6. Dissemination

The support resources were disseminated on the website https://segundasvictimascovid19.umh.es/p/inicio.html [22]. As an outcome measure, visits to the project website were monitored as a proxy indicator of the impact successfully achieved. The resources that were proposed to support practitioners were agreed to be revised as the impact curve bent.

#### 2.4.7. Lessons learned

Participants shared their experience in developing, disseminating, and implementing these interventions. They participated in successive rounds using e-mail to redefine them and establish lessons learned. The consensus among the core group members was obtained once the saturation of the ideas was achieved.

#### 2.4.8. Conflicts of Interest

None of the members of the working group had any significant conflicts of interest. Decisions were made independently and without influence or interest from outside the public service.

## 3. Results

### 3.1. Literature Review

A total of nine references describing the experience of health professionals caring for patients with COVID-19, three reviews studies healthcare workforce distress in previous outbreaks, and two studies regarding professionals’ reactions after caring for victims of natural disasters were identified (Table 1). Supplementary File S1 presents two summary tables. The first table describes the objectives, number of participants and design of each study on the impact of COVID-19 on healthcare professionals. Supplementary File S2 shows the instruments used to measure the impact on professionals and the results obtained.

Managing the uncertainty of caring for patients whose condition is not well understood, the fear of becoming infected and then infecting loved ones, or the reluctance to seek help have been described in previous studies and among professionals in China during the first outbreak of the new coronavirus. Also, the feeling of being rejected and stigmatized by neighbors due to fear of infection and doubts about whether they have sufficient preparation and training to face this situation was mentioned. Anxiety, affective symptoms, feeling exhausted, burnout, insomnia, increased aggressiveness in interpersonal relationships, loss of interest in daily activities, and psychosomatic symptoms are frequent (up to a quarter of the professionals). In general, nurses report more significant distress than physicians. Feeling responsible for patient outcomes (sense of involvement in the task), more even leadership styles, and receiving continuous feedback from managers on the outbreak’s evolution and how things are going help minimize the impact. 

### 3.2. Field Study

The qualitative study identified problem situations in the different participating Spanish hospitals. Three main categories were coded: organization, human and material resources; training and information gaps; and individual affective response. Following these categories, interventions were designed in six areas Organization, Human and Material Resources, Human Factor, Environmental and pandemic-specific stressors, Fear or Panic Reactions, Critical Decision-Making Regarding Health Care Issues, and Post-Crisis.

### 3.3. Interventions

A set of 16 interventions were proposed. These interventions have proven effective in situations sharing some similarities but are, in essence, different from those motivated by the SARS-CoV-2 pandemic. These actions have been established with the consensus of the work team (composed of physicians from other specialties, nurses, clinical psychologists and those responsible for quality and patient safety). The situation’s urgency has not allowed us to establish an empirical analysis of its effectiveness or identify which measure elements are keys to the outcome.

The first group of interventions aimed at strengthening work morale (Table 2). The second group addressed the high number of professionals who had to remain at home in isolation until the epidemiological discharge (Table 3). These staffs were willing to collaborate and support their colleagues but had not been trained to do so. The third group of interventions provides professionals with resources to deal with the emotional overload caused by caring for patients with COVID-19 in extreme situations and preventing severe anxiety and affective syndromes (Table 4). The fourth group of interventions sought to strengthen the role of middle managers, who are indispensable leaders in organizing the response to the challenges posed by this health crisis (Table 5).

**Table 1:** Summary of results of the pragmatic literature review of studies on stress and affective reactions of professionals who attended patients with COVID-19 compared to previous outbreaks.

COVID-19 Pandemic [1,2,23,24,25,26,27].	Other Previous Outbreaks [28,29,30]
Healthcare professionals were reluctant to seek psychological help or to participate in group or individual sessions provided to them to cope with acute stress, despite clear signs of acute stress or panic reactions.	Frontline workers felt helplessness, extreme vulnerability, uncertainty, life-threatening, and increased job stress during the initial phase of disease outbreaks.
For a large number of the professionals, the main concern was the fear of infecting their families upon returning home.	Avian influenza outbreak studies: The majority of the primary care physicians expressed concerns about their family members being at risk of infection with avian influenza because of their jobs. Frontline workers experienced more prejudice from others, perceived a higher risk that they or their family members would contract or die from the infection, and felt stigmatized and rejected by their neighbors.
The lack of means to prevent contamination between professionals was one of the leading causes of acute stress.	Lack of adequate training, peer support, and social support were risk factors for all adverse outcomes following public health disasters.
Many were dismayed at not knowing how to treat patients when they, or their families, did not accept being isolated in the hospital or following the instructions given to them.	Quarantine was the factor most strongly associated with acute stress disorder, feeling stigmatized, considering quitting work, and impaired job performance. Job stressors included a commitment to the ability to do one’s job and a lack of work-related control, including involuntary conscription.
Professionals called for more frequent breaks, guidance on dealing with the emotional problems of COVID-19 patients and their families, and referral to mental health resources.	Inadequate psychological support from employers, inadequate insurance/compensation, frontline staff feedback not reaching managers, and poor sense of team were reported as risk factors for poor mental health.
The following helped to reduce stress: providing food and other products to make daily life easier, encouraging them to talk to their families during the workday, relieving their tension, their families’ concerns and the pressure of professionals among them, providing hotels to stay in and training courses on how to deal with acute stress.	Factors that were positively correlated with HCWs’ willingness to care for patients with SARS: having a positive attitude toward caring for their patients, feeling professional obligation as HCWs to care for their patients, perceived subjective standards (i.e., from superiors), had more significant contact with SARS patients, having self-efficacy, thinking they had resources to care for SARS patients, knowing SARS, perceived institutional measures (i.e., protective facilities or equipment such as those used in university hospitals) to be adequate. Other measures identified in the studies included the use of PPE (i.e., masks, gowns, gloves, and goggles) in accordance with infection control protocols, self-monitoring for signs and symptoms of SARS, temperature control of all staff and visitors, restrictions on visitors, and cancellation of outpatient visits. The precautions were considered effective in limiting the spread of SARS and adequate to prevent it.

Other two preprint manuscripts were reviewed: Jiang N, Jia X, Qiu Z, Hu Y, Yang F, Wang H, et al. The influence of health beliefs on interpersonal loneliness among frontline healthcare workers during the 2019 novel coronavirus outbreak in China: a cross-sectional study. SSRN. 2020. doi: 10.2139/ssrn.3552645; and Dai Y, Hu G, Xiong H, Qiu H, Yuan X. Psychological impact of the coronavirus disease 2019 (COVID-19) outbreak on healthcare workers in China. medRxiv Posted 6 March 2020 [Preprint]. DOI: 10.1101/2020.03.03.20030874.

Situations identified as the main causes of distress for the healthcare professional during the outbreak are summarized in Box 1. Frequent problem situations were classified into three categories (Box 1).

Box 1 **Frequent problem situations identified in the first outbreak.**

**ORGANIZATION, AND HUMAN AND MATERIAL RESOURCES:**

  Receiving rapidly changing instructions (unclear task assignment), coupled with inconsistencies in the chain of command. Infra-utilization or misuse of all health resources due to lack of coordination. Lack of epidemic management protocols.

  Realizing that patients with other pathologies other than COVID-19 do not receive the care they need.

  Mandatory triage systems and forceful rash decisions are reserved for situations of major disasters with a high component of ethics conflict. There has been a transfer of decisions with ethical content to professionals.

  Reduced human resources due to the loss of professionals with a high risk of exposure.

  Dissolution of consolidated work teams.

  Scarcity of adequate materials and supplies (according to centers and services) to alert to or provide protection against biological risk (masks, spectacles, tests, among others) and disruption of the supplies chain (according to centers and services).

**TRAINING AND INFORMATION GAPS:**

  Working in unusual healthcare environments without receiving appropriate training, usually due to abrupt incorporation into or transfer to new and/or more complex environments.

  Lack of training and confusing information on protective measures for the professional’s family members of professionals and on measures to isolate patients in the home to prevent contagion.

**INDIVIDUAL AFFECTIVE RESPONSE:**

  Irritability, especially when imprudent behavior was observed in patients or their companions and when facing fatigue-induced failures, emotional overload, or inability to concentrate on the task.

  Feeling powerless when patients in fear of being sick with COVID-19 are isolated and, in some cases, die in solitude.

  Being emotionally overwhelmed without daring to share this experience with others.

  Fear of infecting the family and/or relative and close friends.

**Table 2:** Interventions aimed at strengthening work morale.

Intervention	Level of Evidence *	References
**Avoiding information blackout.**		
Purpose. To maintain initiative and transparency in the communication strategy during the crisis period. To reduce the impact of false news, which are frequent during crises. Method. Routinely and systematic reporting of scientific, technical, and organizational developments and updates, justifying procedures changes, reporting results in response to the pandemic, including deaths from SARS-CoV-2. The need to disseminate scientific advancements was emphasized, and news showing the responsiveness of professionals to the pandemic. To achieve a more significant impact, the participation of work team members was highlighted (e.g., to explain the correct use of personal protective equipment, how to report isolation conditions to patients and family members, how to deal with acute stress)	1+	Berrouiguet et al. [31] MacLeod [32] Quinn [33]
**Uniformity of criteria**		
Purpose. To facilitate understanding information in an environment with constant protocol updates and disparate instructions that cause mistrust and undermine work morale. Method. Establishing templates for the messages issued by the centre management that should be based on the “more is less” principle. Communication channels should be uniform and the message format designed to facilitate rapid understanding.	2+	Edworthy et al. [34]

* Scottish Intercollegiate Guidelines Network. Forming guideline recommendations. In: SIGN 50: A guideline developers’ handbook: Edinburgh: SIGN; 2008.

**Table 3:** Interventions aimed at better equipping isolated personnel to be of service.

Intervention	Level of Evidence *	References
**Mentoring new professionals in the care team**		
Purpose. The high number of health professionals in home isolation drastically reduced the number of people facing the pandemic to cushion the impact of the high number of health professionals. Method. Assigning a mentor (home isolation professional) to new professionals who join. Enable mentor queries via text messages, audio recordings and applications such as WhatsApp or Google Hangouts. The mentor could give training “pills” on specific aspects.	2−	Raiman et al. [35]
**Ensure the responsiveness of professionals**		
Purpose. To maintain the work and emotional performance of the health personnel in home isolation. Advice (teleconference) of the services of preventive medicine and occupational health for resolution of doubts. Method. Maintain contact and inform the personnel in isolation of the situation in the centre so that their reinstatement is easier.	4	Zhang et al. [36]

* Scottish Intercollegiate Guidelines Network. Forming guideline recommendations. In: SIGN 50: A guideline developers’ handbook: Edinburgh: SIGN; 2008.

**Table 4:** Interventions to provide professionals with resources to deal with the emotional overload caused by caring for patients with COVID-19.

Intervention	Level of Evidence *	References
**Briefings**		
Purpose. Increasing the capacity to face the pressure to which the professionals were submitted with specific orientations on a working day, especially in the case of newly instated in the assistance team. Method. Holding short group sessions (approximately 15 min) before the beginning of the workday and the service professionals’ participation in that shift. Daily breaks are destined to share essential information and generate a climate of confidence and encouragement to face the shift.	2+	Klomp et al. [37] Bethurne et al. [38]
**Protecting the family**		
Purpose. To reduce the impact of the fear of infecting housemates when they return home. Method. Providing instructions on how to act when health professionals arrive home. Arrange for hotel rooms to offer alternatives to returning home and maintaining contact with family members. Providing recommendations on how to keep the relationship with the children in this situation.	4	Chen et al. [1]
**Recovery from physical and emotional fatigue**		
Purpose. To reduce the subjective sensation of fatigue experienced by professionals exposed to highly demanding situations through scheduled short breaks during the workday. Method. Establishing regulated recovery times (of about 5–7 min two or three times per shift) during the day. At the same time, rest areas were to be located in the centre.	2−	Scholz et al. [39]
**Psychological first aid**		
Purpose. To provide professionals with resources to deal with the emotional overload and extreme stress involved in caring for patients with severe COVID-19. Method. Raising awareness among professionals of the need to care for themselves to provide adequate health care to others. The SARS-CoV-2 Emotional Overload Scale was designed and validated. Second, psychological first aid was recommended by designing brief interventions based on:	3	Ripp et al. [40] Greenberg et al. [41] Li et al. [42]
Abdominal breathing	2++	Perciavalle et al. [43] Kim et al. [44]
Jacobson’s progressive muscle relaxation	1+	Asghari Jafarabadi et al. [45]
Mindfulness (STOP technique)	2++	Ducar et al. [46]
Positive psychology (positive notes)	1+	West et al. [47]
Specialized psychological support hotline	1+	Castro et al. [48] Coughtrey et al. [49]
**Humanization of care**		
Purpose. To offer guidance to professionals on how to help families of deceased COVID-19 patients in their grief. Method. Provide orientations to help professionals identify the emotional needs of relatives of patients who had died or were in the moments before their death. It was possible to establish telematic means of communication that would provide emotional closeness and help the relatives initiate mourning in a less traumatic way.	3	Wallace et al. [50] Pattinson [51]
**Emotional overload recovery**		
Purpose. To deactivate the emotional overload before the end of the day. Method. Defusing at the end of the shift in services with extreme situations of emotional overload and favoring a catharsis of the emotional reactions experienced during the shift before returning home. Helping to prevent the symptoms of emotional exhaustion and the complex of sensations, mood swings and psychosomatic symptoms that accompany it.	3	Bohström et al. [52]

* Scottish Intercollegiate Guidelines Network. Forming guideline recommendations. In: SIGN 50: A guideline developers’ handbook: Edinburgh: SIGN; 2008.

**Table 5:** Interventions to strengthen the role of middle managers.

Intervention	Level of Evidence *	References
**Leadership of middle management**		
Purpose. To emphasize the role of middle managers and strengthen their leadership by conveying information and listening to the concerns of their teams Method. To promote their ability to communicate effectively with the care team, reinforcing initiative, transparency of information, equity, agility, and realism in their communication style.	2−	Pons Verdú et al. [53]

* Scottish Intercollegiate Guidelines Network. Forming guideline recommendations. In: SIGN 50: A guideline developers’ handbook: Edinburgh: SIGN; 2008.

The Ser+Contra COVID app was externally evaluated and accredited with the Andalusian Health Quality Agency’s Healthy App distinction [54]. Finally, on 9th and 23rd April 2020, the website and the resources for coping with acute stress were subsequently translated into English and Portuguese, respectively [22].

### 3.4. Barriers and Facilitators

At first, there was resistance among professionals to recognize their personal experience, which, together with the lack of information on identifying signs of acute stress and dealing with these situations, hindered access to support resources. As a result of the daily pressure experienced in the centers and the effect of the media and social networks drawing attention to the workload and acute stress reactions among healthcare professionals, this situation gradually changed.

The factors that may have facilitated the implementation of the recommendations were mainly motivated by the alarming situation experienced in the centres. The dissemination of the website in social networks, press, TV and radio also helped. Dissemination employing successive webinars (national and international) and the information on the website of second victims of patient safety incidents, which brings together some good news sections and the active role of professionals on the social networks, contributed to its dissemination.

### 3.5. Website Visits

Between 31 March and 13 April, 43,907 visits were recorded. In the following months, the developed resources were also applied in several Latin American countries.

## 4. Lessons Learned

### 4.1. Shifting Social Support

At the time when the support resources for professionals were designed, society considered them to be heroes. Once the confinement ended, as the incidence of the first outbreak decreased, this social valuation dissipated, and the number of disputes over the care (or lack of care) given to COVID-19 and non-COVID-19 pathologies increased. Although we contemplated this possibility among the precautions for the post-crisis, we did not identify appropriate resources to deal with this situation.

### 4.2. False Expectations

The prediction of a return to normality announced by the health authorities has not been fulfilled. Unlike other outbreaks (avian influenza, SARS-CoV, Ebola), the COVID-19 pandemic continues over time with different outbreaks and in very different ways depending on the territory. This causes the adaptation responses of professionals to change, and new affective reactions may appear, together with burnout and disenchantment with the organization. Adding to the risk of infection for the professionals’ families and themselves, this circumstance also makes the situation different concerning other situations such as multiple accidents, natural disasters, or terrorist attacks with numerous victims. The resources we proposed were selected for the specific situation experienced in March and April in healthcare centres in Spain and may not have the same usefulness at other times with a different social environment and work morale in healthcare centres.

### 4.3. Unidentified Factors

We did not identify in our initial analysis that administrative bureaucracy would negatively affect work morale. Even though protocols and processes in the clinical setting have been modified in a matter of hours, organisational processes have not had the same agility of adaptation, negatively impacting professionals’ confidence in the health system.

Departments that require specific training, such as critical care units, that under normal conditions we find a majority of highly qualified professionals (A), some professionals with average experience (B) and few new professionals with no previous experience (C). At the beginning of the outbreak and because of the number of professionals initially infected (in Spain, the volume of infected professionals exceeded 25% of the total number of COVID-19 patients [55]) or in isolation due to close contact, it was necessary to form mixed teams made up of professionals from the three groups (A, B and C). Considering that those from groups A and B would act as leaders with those from group C. However, those of group B who, under normal conditions, could assume this task, in this case, were overwhelmed and were probably the group that bore the most significant burden of acute stress, without our having foreseen specific interventions for this situation.

At the end of their shift (especially if they had to work double shifts), the professionals wanted to leave the center as soon as possible. This aspect was not adequately assessed at the beginning. No activities outside the healthcare centers were devised.

One of the support resources that we have identified later is that the professionals value positively to see responsible behaviors to avoid infection among the citizens. This has become more evident in successive waves.

### 4.4. Communication Barriers

There has been a continuous oversaturation of information, both in quantity and diversity of sources and with too much “noise”. Fake news appeared quickly, and although we cannot determine its effect on professionals, this work could be considered in the future by occupational health services as another disruptive factor. In addition, the increasing number of negative news in all media influenced the morale of professionals, an aspect that we did not initially take into account.

Information oversaturation also stemmed from the amount of information that was quickly generated on the impact of COVID-19 patient care on the wellbeing of professionals and a growing and disorganized proliferation of suggestions and recommendations. In our case, we sought to simplify the information (e.g., through infographics) since we understood that an expansion of news and information channels produces misinformation.

### 4.5. Success Factors

Uncertainty and complexity have continued, and it has been necessary to accept the continuous changes in the protocols and rules of action. Even months after the pandemic, this situation persists, and the teams have learned to tolerate uncertainty and make decisions in this context. This aspect was not initially appreciated and is likely to have been one of the success factors of the teams in dealing with the pandemic.

Keeping staff informed about the evolution of the pandemic, the availability of equipment and resources, organizational changes and the reasons for them have been another success factor.

In this crisis, we have seen teams that have been able to draw strength from where there seemed to be nothing, overcoming adversity. Clinical leadership has made a difference in the organizational and technical capacity of healthcare organizations. This aspect requires further attention in the future to leverage the advantage it represents for healthcare organizations, probably also after this healthcare crisis is over.

Organizational flexibility and the versatility of the different specialties to perform tasks other than their usual ones (e.g., allergists in occupational health, surgeons in emergency medicine) should be assessed to see whether it has been a protective factor for professionals to the solidarity it represents.

### 4.6. Adaptation to the Urgency of the Moment

The need for information has meant that other high-risk sources have replaced traditional sources of evidence due to the lack of critical evaluation (e.g., articles without prior evaluation, etc.). The use of non-conventional means of scientific communication has been indispensable, although it entailed a greater risk for patient safety.

Traditional measures to determine the effectiveness of interventions have given way to others based on visits to a web page, downloads of health apps, among others. The time available was too short to use traditional outcome assessment models. In this sense, methods already relegated, such as action-reaction, have regained their usefulness at the onset of this health crisis.

## 5. Discussion

The combination of social alarm, services over-saturation, scarcity of resources, and torpid evolution of patients by COVID-19 has made health professionals suffer acute stress, limiting their wellbeing and, in some cases, moral damage [3,4,41].

In Spain, during the first outbreak a large number of health professionals experienced confusion, frustration, fear, hopelessness, and doubts regarding their abilities and those of the healthcare system [17]. In some cases, anxiety syndromes and psychosomatic conditions were developed [3,56]. These experiences were similar to those described in other countries [1,2,23,24,25,26,27] and confirm the need to address these reactions to keep the pandemic response capacity operational. 

In addition to the already known concerns regarding mechanical ventilator and medication shortage, and the fear of infecting family members, this study highlights other issues. Primary care has seen its work dynamic broken and has had to adapt quickly to a completely different care environment for which it was not prepared. The mortality among older adults living in nursing homes has exceeded all estimations [57]. The number of professionals infected has also been of great concern (around 25% of COVID patients were healthcare workers [55]). Uncertainty due to changes in the procedure for time off work of health professionals suspected of suffering COVID-19. The triage personnel in extreme conditions and the inability to respond to the needs of patients and their families, especially in the case of death in isolation. These difficulties have posed disproportionate challenges for which the professionals had not been trained. 

The acute stress reactions that have been registered have been considered that could progress to anxiety-depressive syndromes and post-traumatic stress [58]. Nevertheless, it is not only the impact on individuals that needs to be addressed. Work teams have also changed and have had to learn to cope as a group with the challenge caused by SARS-CoV-2. Although this aspect has received less attention [14,59], our experience is that appropriate dynamics in managing the emotions and experiences of professionals, together with clear instructions, information sharing, listening skills and flexibility in organizational decisions, have contributed to better coping with the distress.

Secondary prevention is crucial to guarantee enough workforce to deal with the return to normality [10]. This will be particularly relevant in the coming months, when all patients who had their due course of treatment interrupted will require attendance and when new pathologies generated by the period of confinement and those typical of the sequelae of COVID-19 arise. The health professionals who will have to take on this task are the same ones who have now faced the worst moments of the pandemic. The alarming growth in the number of discouraged professionals has led to the creation of this workforce.

These recommendations have supported frontline professionals caring for COVID-19 patients, maintaining labor morale and reducing the immediate and foreseeable medium-term emotional impact and overload. The number of visits to the website exceeded 110,000 by the end of 2020. This approach supporting frontline practitioners has been urgently needed and has been similar in many countries [60]. The successive waves of the pandemic have highlighted those periods of lower incidence should be used to strengthen these professionals’ resilience. We cannot forget that it is also necessary to work on post-crisis planning [61].

The conventional pattern when a catastrophe occurs is that after the impact, work morale is rebuilt, and emotional equilibrium is restored as the source of stress ceases [62]. This was not the case. The situation of professionals who have participated in the care of victims of natural disasters, terrorist attacks, or major railway or aviation accidents is quite different from the circumstances of this SARS-CoV2 pandemic [63,64]. However, we did identify similarities with what happened in other previous infectious outbreaks, although the lessons learned in those cases have not always been taken into account in the current COVID-19 crisis [65,66,67]. The first information was available until other results began to be disseminated from the experience in Wuhan hospitals [27].

Another relevant aspect, to which less attention has been given, is that the professionals’ confidence was a necessary factor to boost the morale of society at a critical moment. The population needed to see the professionals in place and control of the emergency. Our group identified this aspect in the early phase of the pandemic when the decision was made to quarantine the population.

These interventions have been proposed by the centers participating in this tasks force, reflecting their experience. Their feedback has been used to improve recommendations regarding this matter. The results of this proposal were disseminated on a specific website [68]. 

The main limitation of this study is that the effectiveness of each of these interventions for the purpose for which they have been applied has not been contrasted through experimental or quasi-experimental studies. The quickness of extraction from data sources may have limited access to other relevant references. In this context, a detailed analysis was spared for the immediacy of decision-making. Although coinciding with those described in other countries, the identified stressors were very uneven in intensity in the different territories, making it difficult to assess whether a generalist intervention would be appropriate. 

Undoubtedly, lessons have been learned as the pandemic progresses, but we still have work to do. There are personal differences that have led healthcare workers to desert their jobs or even decide to work in the front line of the pandemic; possible underlying reasons are not yet known. Health systems and, in particular hospitals are very hierarchical structures, as has already been exposed in other studies [69]. It is necessary to elucidate the decisions and motivations that have led this hegemony to become more flexible and promote multidisciplinary work. Critical decisions were made during this pandemic, how these stressful situations impacted policy makers and senior management needs to be considered. Few studies have included managers and middle management, but they highlight interventions similar to those proposed in this work. Other authors have identified six thematic areas for efficient leadership (1) Effective communication and transparency; (2) Prioritizing their health and safety; (3) Employee scheduling considerations: autonomy, assignment support and respite; (4) Appreciation- financial and nonfinancial; (5) Showing up and listening and (6) Stepping up with resources as key elements in caring for healthcare professionals [70]. Studies on community coping with catastrophic situations have described that the psychological response evolves in impact, heroic (intensification of efforts), honeymoon (optimism) and disillusionment (fatigue) and restoration (recovery) phases [71]. As we are still coping with the successive waves of COVID-19, these phases have been modified and changed what we could have expected to occur. In this sense, more research is still needed to respond to future pandemics and other unanticipated crises to strengthen health care systems as a whole.

As Shanafelt et al. [2] have pointed out, health care professionals expected that organizations in which they work would hear them, protect them, prepare them, support them and care for them. In previous infectious disease outbreaks, many of the conditions that have been experienced with COVID-19 were identified. However, those lessons do not appear to have been effectively incorporated into planning. We will learn more about the variability in how these recommendations have been applied and their usefulness in mitigating the impact of COVID-19 on the morale of teams and individual professionals in the following months. In the meantime, we have some lessons learned from this and other pandemics that should be useful in the future.

## 6. Conclusions

The emotional impact of this health crisis has been in some respects like that of previous outbreaks. The fear of becoming infected and/or infecting family members, the resistance to receive help, the reluctance to ask for support or the anxiety and affective disorders that have been experienced have many similarities. On the other hand, the moral damage caused by the loss of patients due to the scarcity of resources or the value of peer support in this new crisis has some different characteristics from previous outbreaks. 

The experiences of previous outbreaks have not led to the adoption of interventions to train professionals to face new crises. In this pandemic, some of the experiences have been repeated. This situation must be avoided from now on.

The involvement of occupational health departments has been essential, and in our case, it has been especially useful to incorporate their knowledge of the experience being lived in the centers. In the recovery phase, these departments should be reinforced to pursue improvements in occupational wellbeing. 

In this outbreak, we have observed that the interventions that have encouraged more participative leadership styles, the organizational culture that facilitates interdisciplinary teamwork and the feedback of information provided by middle managers and supervisors have been positive in minimizing its impact. The focus of supporting interventions should not be only on workers as individuals but also on the team in which they are integrated. In our case, interventions were proposed to strengthen the morale of the group. This aspect could be reinforced to face new health crises in better conditions.

The briefing techniques and those reinforcing peer support were the most accepted by the professionals. Feeling that the institution is concerned about their welfare is another factor that favored facing the impact of the pandemic. Examples of high demanding interventions were recommendations related to protocols when arriving home to avoid contagion. Helping isolated patients maintain contact with their families. And how they could maintain contact with their young children when they chose to live in a hotel for professionals or when the professional was in isolation due to infection or close contact.

Although the COVID-19 pandemic has a more devastating effect than other recent outbreaks, there are common stressors and lessons learned in all of them that we must draw on to increase our capacity to respond to future healthcare crises.

## Data Availability

Not applicable.

## Supplementary Materials

The following are available online at https://www.mdpi.com/article/10.3390/ijerph182212010/s1.
