# Supplementary material for: Addressing Acute Stress among Professionals Caring for COVID-19 Patients: Lessons Learned during the First Outbreak in Spain (March–April 2020)
Source: Int J Environ Res Public Health. 2021 Nov 16;18(22):12010. doi: 10.3390/ijerph182212010 (PMC8624221; doi:10.3390/ijerph182212010)
Supplement: Supplementary file 1 [file ijerph-18-12010-s001.zip › Suppl file S1.pdf]

Supplemental file S1

Authors, year	Objective	Disease	Number of included works	Participants	Main findings
(Koh et al., 2011)	To analyze the perceptions of healthcare workers regarding the risk of exposure to emerging acute respiratory infectious diseases and the perceived effectiveness of the strategies used to deal with them.<br>Review provided answers to the following questions:<br>**1** How do HCWs practicing in hospitals and community healthcare settings perceive the risks of being exposed to recently EARIDs?<br>**2** What are the socio-cultural, psychological, attitudinal and environmental factors influencing the HCWs' risk perceptions?<br>**3** What are the individual strategies adopted by HCWs when exposed to EARIDs?	Emerging acute respiratory infectious diseases (EARIDs): severe acute respiratory syndrome (SARS), the avian influenza A/H5N1 virus and the influenza A/H1N1 virus.	14	Healthcare workers'	**How do HCWs practicing in hospitals and community healthcare settings perceive the risks of being exposed to recently EARIDs?**<br>*Health risks*<br>SARS outbreak studies: Most HWs perceived high risk of infection by the virus, expressed fear of becoming infected, little control over the possibility of becoming infected, and little chance of survival if infected.<br>Avian influenza outbreak studies: The majority of HCWs perceived, because of their jobs, to have a greater risk of exposure to the disease and a higher risk of infection. They were more afraid of the pandemic influenza and of falling ill with the disease.<br>*Social risk*<br>SARS outbreak studies: Data ranged from 28% to 60% of respondents who indicated that their friends and neighbors avoided them. One-third of respondents believed that people avoided their relatives for fear of contracting SARS. Health workers reported fear of being stigmatized and shunned by others. However, it was not ascertained whether they had experienced stigmatization.<br>Most participants were concerned about transmission to family, friends, and colleagues, and, likewise, believed that their loved ones were equally concerned about the possibility of becoming infected with the disease through exposure to WS. In one study, 38% reported lifestyle changes by avoiding interaction with friends and family and avoiding public spaces.<br>Avian influenza outbreak studies: The majority of the primary care physicians surveyed from both private and public healthcare settings perceived that others would avoid them and their family

	**4** What are the key organizational, environmental and individual factors influencing their use of the strategies?<br>**5** What are the organizational strategies implemented and how do HCWs perceive the effectiveness of the implemented organizational strategies?				members. They also expressed concerns about their family members being at risk of infection with avian influenza because of their jobs.<br>*Acceptance of risks*<br>Most of HCWs respondents accepted that the risk of becoming infected with SARS (70% and 64%) and avian influenza (82.5% and 75%) was part of their job.<br><br>**What are the socio-cultural, psychological, attitudinal and environmental factors influencing HCWs' risk perceptions?**<br>SARS outbreak studies: The results showed that having higher risk perceptions were associated with being female, staying with children, having higher IES scores, indicating emotional distress, perceiving that their personal or family lifestyle was affected, perceiving feelings of stigmatization by the public, personally knowing someone, especially colleagues, who had contracted SARS, having previous exposure to infectious agents, and/or perceiving the risk of death from SARS to be approximately 5% or more. On the opposite, older HCWs had a lower perception of risk.<br>Organizational factors: Nursing staff, compared to medical staff, who worked in non-university hospitals and/or who worked during the early phase (reaction phase) compared to those who worked in the late phase (repair phase) of the SARS outbreak, were significantly more likely to have higher risk perceptions toward SARS. HCWs who worked in managerial or supervisory positions, who had little or no exposure to SARS patients per week, who worked in hospitals not affected by SARS, and who perceived that they had organizational safeguards (i.e., protective equipment, clear infection control policies and protocols, infection control training, and other institutional policies and measures in place) had lower risk perceptions.<br><br>Avian influenza outbreak studies: HCWs who had families with fear of infection and/or worked in tertiary hospitals (TH), compared to

					those who worked in community hospitals (CH), had significantly higher risk perceptions. Elements that reduced HCWs' fear were having knowledge about the impact of pandemic influenza, receiving timely updates from the healthcare organization, perceiving that their job role is part of the organization's response to avian influenza, perceiving that they are familiar with the response specific to their role, and having the ability to communicate risk effectively and/or perceiving that their families are protected.<br><br>**What are the individual strategies adopted by HCWs to manage their risk perceptions when exposed to EARIDs and what are the key organizational, environmental and individual factors influencing their use of these strategies?**<br>*Behavior towards patients*<br>SARS outbreak studies: HCWs' negative behavior towards their patients was found to encompass avoiding and unwillingness to care (91.7%) and resigning and/or finding new jobs (27%). In contrast, other studies showed that only a minority of HCWs felt that they should not care for SARS patients (12.2%) or intended to resign due to perceived risks (25.9%).<br>Avian influenza outbreak studies: Most HCWs were willing to report to work and care for infected patients; only 10% considered looking for another job as a result of the risks.<br><br>*Factors influencing HCWs' behavior towards patients.*<br>SARS outbreak studies: HCWs who were nurses, were female, were younger (i.e., less than 35 years), had fewer years of experience (i.e., less than 8. 3 years), had a fear of SARS, perceived greater likelihood of dying from SARS than from cancer, perceived stigmatization as a consequence of their work, perceived to have a greater workload, and worked in non-university hospitals were significantly more likely to exhibit avoidant behavior.

					Factors that were positively correlated with HCWs' willingness to care for patients with SARS: having a positive attitude toward caring for their patients, feeling professional obligation as HCWs to care for their patients, perceived subjective standards (i.e., from superiors), had greater contact with SARS patients, having self-efficacy, thinking they had resources to care for SARS patients, having knowledge about SARS, perceived institutional measures (i.e., protective facilities or equipment such as those used in university hospitals) to be adequate.<br>Avian influenza outbreak studies: Reveal similar results. HCW who were afraid, who worked in private healthcare settings and/or in tertiary hospitals were significantly less disposed to care for patients compared to those who worked in public settings and those who worked in community hospitals.<br>In contrast, factors found to be correlated with HCWs' willingness to care for infected patients and/or report to work were involvement in religious activities, perception that infection control measures and equipment were sufficient, perception that their role was an important part of the organization's response to the pandemic, and perception of ability to effectively communicate risk.<br><br>*Compliance to preventive measures*<br>Most respondents reported little difficulty in complying with the measures (72%) and complied with the measures implemented during SARS (92%).<br>Mask use was cited as the most annoying precaution (85.4%), a source of physical discomfort (92.9%) and affecting their communication (47%).<br>Nurses perceived greater difficulty in complying with preventive measures compared to physicians. The opposite occurred with HCWs who had more work experience.<br><br>**What are the organizational strategies implemented and how do HCWs perceive the effectiveness of these strategies?**

					SARS outbreak studies: Measures identified in the studies included the use of PPE (i.e., masks, gowns, gloves, and goggles) in accordance with infection control protocols, self-monitoring for signs and symptoms of SARS, temperature control of all staff and visitors, restrictions on visitors, and cancellation of outpatient visits. The precautions were considered effective in limiting the spread of SARS and adequate to prevent it.<br>Avian influenza outbreak studies: Similar organizational measures. Most HCWs reported receiving training in infection control, use of PPE, being informed of the hospital's outbreak preparedness plan, and being advised to be vaccinated against influenza. As a result, the majority of PS respondents felt that they and their organizations were prepared.
(Naushad et al., 2019)	1.- To determine the psychological impact of disasters on medical responders<br>2.- To identify the possible risk factors associated with psychological impacts on medical responders	Disaster: disaster, natural disaster, man-made disaster, flood, tsunami, earthquake, landslides, calamity, cyclone, hurricane, tornado, severe acute respiratory syndrome (SARS), SARS epidemic, swine flu outbreak, HlNl outbreak, HlNl influ-enza, Middle East respiratory syndrome (MERS), MERS outbreak, Middle East respiratory syndrome outbreak, corona virus infection, Ebola outbreak, terror attack, bomb-ing, suicide bombing, hostage crisis, kidnapping, air	28	Doctors 21; nurses 21; Paramedic, disaster medical personal 7; allied health staff 4; admin staff 3; non-medical responders 12	Studies focused on hospital staff exposed to patients with SARS virus infection, including doctors, nurses and ancillary healthcare workers who worked in emergency rooms, intensive care units, respiratory medicine units, medical wards and SARS isolation wards. This included hospital care workers exposed to patients from the 2003 SARS outbreak in Taiwan, in Canada, in Hong Kong, in Singapore; physicians, nurses and ancillary healthcare workers exposed to patients from the 2009 HlNl flu outbreak in Greece; and physicians exposed to patients from the MERS outbreak in Saudi Arabia.<br>Psychological impact:<br>The prevalence of Posttraumatic Stress Disorder ranged from just under 3% to 20% (mean 14%).<br>The prevalence of depression varied from approximately 10% to 74% (median 35%).<br>The prevalence of anxiety ranged between approximately 14% to 77% (mean 37%).<br>The prevalence of somatic complaints ranged between approximately seven percent to 48% (mean 25%).

		crash, mass shooting, bus bombing, train bombing, World Trade Center (WTC) attack, war, Gulf war, Arab war, Syrian war, Middle East war, Lebanon war, Israel war, Arab spring.			The prevalence of posttraumatic distress among HCWs ranged approximately between five percent to 45% (mean 27%).<br>Prevalence of sleep disturb-ance ranged between 11% to 52% (mean 28%).<br>The prevalence of bumout among HCWs 13-26 months after the 2003 SARS outbreak was 30%.<br><br>Correlates of Adverse Psychological Outcomes<br>Male gender had a higher risk of developing posttraumatic stress in health care workers. When analyzing subgroups of healthcare workers, nurses were found to have greater psychological problems compared to physicians: higher levels of post-traumatic stress and depression and higher levels of emotional distress and anxiety.<br>Lack of adequate training, peer support, and social support were shown to be risk factors for all adverse outcomes following public health disasters, while years of professional experience were inversely associated. In addition, working in high-risk units (i.e., emergency, ICU, and isolation wards) had a higher predisposition for developing adverse psychological outcomes following public health outbreaks.<br>Maladaptive coping through avoidance, aggressive confrontation, and self-blame contributed to PTSD, burnout, anxiety, and post-traumatic distress in HCWs who cared for SARS patients. Levels of distress and anxiety were higher in the early phase than in the recovery phase of the SARS outbreak. Frontline workers experienced more prejudice from others, perceived a higher risk that they or their family members would contract or die from the infection, and felt stigmatized and rejected by their neighbors.<br>Front-line workers felt helplessness, extreme vulnerability, uncertainty, life-threatening, and increased job stress during the initial phase of disease outbreaks.<br>Other elements, such as lack of communication, educational level, and marital status, correlated with adverse psychological outcomes following public health outbreaks. Increased smoking, alcohol

					consumption, and substance abuse were also observed among healthcare workers following SARS outbreaks.<br>Perceived stress immediately after the disease outbreak has been reported as a risk factor for subsequent psychiatric morbidity.<br>However, the HCWs also had positive psychological effect after the SARS outbreak. Positive responses were reported by 94% of HCWs, including hygiene mindfulness (85%), attention to current events (77%), and hazard awareness (41%).<br>Age, education, marital status, workload, number of children or family members, health and relationship with family, support from family, spiritual beliefs, and religious convictions had no correlation.
(Brooks et al., 2018)	To identify social and occupational factors affecting the psychological wellbeing of healthcare workers involved in the SARS crisis.	SARS crisis	22	healthcare worker, doctor, nurse	***Occupational Factors***<br>*Occupational role*<br>Nurses were more likely to show symptoms of post-traumatic stress disorder, to report elevated stress levels and a greater number of mental problems, to report high levels of distress.<br><br>*Specialized training and preparedness*<br>The studies emphasized the importance of training, either through specialized instruction or previous crisis experience. Healthcare workers who were confident in their knowledge and skills in infection control had lower stress levels than those who felt less prepared.<br><br>*Working in high-risk environments*<br>An association was found between working in "high-risk settings" (usually defined as those with high levels of exposure to SARS patients) with poor mental health outcomes. Staff working in such units were found to report higher stress and avoidance, higher levels of post-traumatic stress symptoms, and higher alcohol consumption and greater post-traumatic stress symptoms than those who did not work in high-risk units. They were also more likely to report fatigue, lack of sleep, health concerns, and fear of social contact during the SARS crisis, along with greater depression

					and anxiety after SARS. Staff who cared directly for SARS patients had higher levels of distress, post-traumatic stress disorder symptoms, emotional exhaustion, stress and distress, intrusive symptoms, hypersensitivity, and concern about stigma than those who did not work directly with patients.<br><br>*Quarantine*<br>Quarantine was the factor most strongly associated with acute stress disorder, feeling stigmatized, considering quitting work, and impaired job performance. It was also associated with greater post-traumatic stress symptoms and higher alcohol consumption. Duration of quarantine predicted anger and avoidance behaviors, and those who were in quarantine for longer showed more adverse effects.<br><br>*Job stress*<br>Job stressors included commitment to the ability to do one's job and lack of work-related control, including involuntary conscription. Associated with emotional distress was compromised ability to do one's own work due to SARS precautions, feeling vulnerable and loss of control, and changes in work.<br>Control over work, in terms of whether the specific role was voluntary or not, had an impact on well-being. Those who were willing to work in SRAG units reported worse mental health than those who were against volunteering.<br><br>*Perceptions of safety, threat, and risk*<br>More confidence in infection control team and procedures predicted less emotional exhaustion and less anger in nurses.<br>Perceived greater personal risk was significantly associated with greater concern for personal or family health, whereas belief that workplace precautionary measures were sufficient was associated with decreased levels of concern; perceived self-risk also predicted post-traumatic stress symptoms.

					Social Factors<br>*Organisational support*<br>In nursing staff, poor organizational support was associated with avoidance and anger.<br>Inadequate psychological support from employers, as well as inadequate insurance/compensation, front-line staff feedback not reaching managers, and poor sense of team were reported as risk factors for poor mental health.<br><br>*Support from friends and family*<br>Higher family support was associated with lower risk of mental health problems in health care workers and lower anxiety in nurses. Low family support before SARS was associated with depression and sleeplessness, whereas poor family support after SARS was associated with anxiety and sleep problems.<br><br>*Social rejection or isolation*<br>Feeling isolated was associated with heartbreak in physicians, nurses, and health care assistants working in the emergency department. Social isolation mediated the association between contact with SARS patients and being a nurse with psychological distress in healthcare workers.<br>Being discriminated against was associated with poor mental health in healthcare workers. Being treated differently by people because of working in a hospital was associated with increased health concern in healthcare workers.<br><br>*Impact on life*<br>Personal or family life affected by SARS was associated with increased personal/family health worries and emotional distress in hospital workers, and avoidance and hyperactivity in healthcare workers.

Table 2.

Objective, participants, design and date of data collection.

Authors	Target	Participants	Design	Data collection
Liu, et al. ([i]2020)	To estimate the prevalence of anxiety among healthcare personnel.	512 healthcare personnel: medical, nursing and hospital administrative personnel	Cross-sectional through questionnaires via WeChat	From February 10 to 20
Dai, Hu, Xiong, Qiu, & Yuan (2020)[ii]	To investigate the risk perception and psychological state of health care workers.	4357: physicians: n=1419; nursing: n=2343; auxiliary: n=437;: support staff: n=158	Cross-sectional by means of questionnaires distributed through a social network	February 3 to 11
Zhu, et al.[iii] (2020)	Assessing the psychological impact on workers at Tongji Hospital in Wuhan.	5062: physicians: n=1004; nurses: n=3417; auxiliaries n=641	Single-center cross-sectional, through online questionnaires	February 8 to 10
Huang, Xu, & Liu (2020)[iv]	To assess the emotional responses and coping strategies of nursing staff and undergraduate nursing students.	802: nursing staff: n= 374; students: n= 430	Cross-sectional, online survey	From February 1 to 9
Jiang, et al.[v] (2020)	To determine the relationship between self-efficacy and feelings of interpersonal loneliness in front-line health professionals.	205: physicians: n=60; nursing= 145	Transversal, from online questionnaires	From February 15 to March 9
Kang, et al.[vi] (2020)	To explore the mental health status of medical and nursing staff and the effectiveness of psychological support.	994: physicians: n=183; nurses= 811	Transversal, from online questionnaires	From January 29 to February 4

Lai, et al.[vii] (2020)	To assess mental health problems and associated factors in healthcare professionals treating patients exposed to COVID-19.	1,257 health care personnel from 34 hospitals: physicians: n=493; nursing= 764	Cross-sectional, survey-based and stratified by region.	January 29 to February 3
Wu, et al. (2020) [viii]	To compare the frequency of burnout among physicians and nurses at the front line of exposure versus those working in their usual positions.	190: 41 medical professionals treating COVID-19 on the front line and 33 on their usual service; 55 nursing staff treating COVID-19 on the front line and 61 on their usual service.	Transversal, from online questionnaires	March 13 to 17
Siyu, et al. (2020). [ix]	Assessing mental health and social support as a coping strategy in healthcare personnel.	5393: physicians: n= 3011, nurses: n= 92, clinical assistants: n= 235, other staff: n= 215	Cross-sectional through questionnaires via WeChat	February 9 to 15

Brooks, S. K., Dunn, R., Amlôt, R., Rubin, G. J., & Greenberg, N. (2018). A Systematic, Thematic Review of Social and Occupational Factors Associated with Psychological Outcomes in Healthcare Employees during an Infectious Disease Outbreak. *Journal of Occupational and Environmental Medicine*, *60*(3), 248–257. https://doi.org/10.1097/JOM.0000000000001235

Koh, Y., Hegney, D. G., & Drury, V. (2011). Comprehensive systematic review of healthcare workers' perceptions of risk and use of coping strategies towards emerging respiratory infectious diseases. *International Journal of Evidence-Based Healthcare*, *9*(4), 403–419. https://doi.org/10.1111/j.1744-1609.2011.00242.x

Naushad, V. A., Bierens, J. J. L. M., Nishan, K. P., Firjeeth, C. P., Mohammad, O. H., Maliyakkal, A. M., Chalihadan, S., & Schreiber, M. D. (2019). A Systematic Review of the Impact of Disaster on the Mental Health of Medical Responders. In *Prehospital and Disaster Medicine* (Vol. 34, Issue 6, pp. 632–643). https://doi.org/10.1017/S1049023X19004874

[i] Liu, C. Y., Yang, Y. Z., Zhang, X. M., Xu, X., Dou, Q. L., & Zhang, W. W. (2020). The prevalence and influencing factors for anxiety in medical workers fighting COVID-19 in China: A cross-sectional survey. Available at SSRN 3548781. 8 marzo medRxiv

[ii] Dai, Y., Hu, G., Xiong, H., Qiu, H., & Yuan, X. (2020). Psychological impact of the coronavirus disease 2019 (COVID-19) outbreak on healthcare workers in China. medRxiv preprint doi: https://doi.org/10.1101/2020.03.03.20030874. 6 marzo

[iii] Zhu, Z., Xu, S., Wang, H., Liu, Z., Wu, J., Li, G., ... & Zhu, S. (2020). COVID-19 in Wuhan: Immediate Psychological Impact on 5062 Health Workers. medRxiv preprint doi: https://doi.org/10.1101/2020.02.20.20025338. 16 marzo

[iv] Huang, L., Xu, F. M., & Liu, H. R. (2020). Emotional responses and coping strategies of nurses and nursing college students during COVID-19 outbreak. MedRxiv, 2020.03.05.20031898. https://doi.org/10.1101/2020.03.05.20031898. 8 marzo

[v] Jiang, N., Jia, X., Qiu, Z., Hu, Y., Yang, F., Wang, H., & He, Y. (2020) The Influence of Health Beliefs on Interpersonal Loneliness among Front-Line Healthcare Workers during the 2019 Novel Coronavirus Outbreak in China: A Cross-Sectional Study. Available at SSRN 3552645. 12 marzo

[vi] Kang, L., Ma, S., Chen, M., Yang, J., Wang, Y., Li, R., Yao, L., Bai, H., Cai, Z., Xiang, B., Hu, S., Zhang, K., & Wang, G. (2020). Impact on mental health and perceptions of psychological care among medical and nursing staff in Wuhan during the 2019 novel coronavirus disease outbreak: A cross-sectional study. March, 1-7. https://doi.org/10.1016/j.bbi.2020.03.028

[vii] Lai, J., Ma, S., Wang, Y., Cai, Z., Hu, J., Wei, N., ... Hu, S. (2020). Factors Associated with Mental Health Outcomes Among Health Care Workers Exposed to Coronavirus Disease 2019. JAMA Network Open, 3(3), e203976. https://doi.org/10.1001/jamanetworkopen.2020.3976 2 marzo

[viii] Wu, Y., Wang, J., Luo, C., Hu, S., Lin, X., Anderson, A. E., ... Qian, Y. (2020). A comparison of burnout frequency among oncology physicians and nurses working on the front lines and usual wards during the COVID-19 epidemic in Wuhan, China. Journal of Pain and Symptom Management. https://doi.org/10.1016/j.jpainsymman.2020.04.008 10 abril

[ix] Siyu, C., Xia, M., Wen, W., Cui, L., Yang, W., Liu, S., ... Lei, W. (2020). Mental health status and coping strategy of medical workers in China during The COVID-19 outbreak. MedRxiv, 2020.02.23.20026872. https://doi.org/10.1101/2020.02.23.20026872 7 marzo
