# Supplementary material for: Addressing Acute Stress among Professionals Caring for COVID-19 Patients: Lessons Learned during the First Outbreak in Spain (March–April 2020)
Source: Int J Environ Res Public Health. 2021 Nov 16;18(22):12010. doi: 10.3390/ijerph182212010 (PMC8624221; doi:10.3390/ijerph182212010)
Supplement: Supplementary file 1 [file ijerph-18-12010-s001.zip › Suppl file S2.pdf]

Supplementary file S2

Measures collected, instruments used and results obtained.

Authors	Measures	Instruments	Results
Liu, et al. (2020)	Anxiety	Zung Self-rating Anxiety Scale (SAS; 1990) Consists of 20 items, scale 1-4 ranging from 20-80 and the higher the score, the higher the degree of anxiety. Responses are classified into 4 response categories: normal (≤49), low anxiety (50-59), moderate anxiety (60-70), severe anxiety (≥70). Internal consistency= 0.66-0.80; Cronbach's alpha=0.87.	The prevalence of anxiety was 12.5%. 10.35% professionals showed mild anxiety,1.36% moderate and 0.78% severe anxiety. Medical personnel who had been in direct contact with infected patients reported higher anxiety scores than those who had not had direct contact (β = 2.33, CI: 0.65 -4.00; p = 0.0068). Similar scores were observed in medical personnel from Hubei province compared to those from other parts of China (β value = 3.67, CI: 1.44 -5.89; p = 0.0013). The variable most associated with elevated anxiety scores was having suspicion of the disease (β value = 4.44, CI: 1.55 -7.33; p = 0.0028).
Dai, Hu, Xiong, Qiu, & Yuan (2020)	Risk perception General psychological state	6 questions about the perceived risk of COVID-19, scored on a five-point Likert scale (1= Very concerned to 5= Not at all concerned). Are you concerned about becoming infected with COVID-19? 2. Are you concerned about infecting your family members with COVID-19? Are you concerned about medical violence? 4. Are you concerned about your front-line colleagues (direct contact with COVID-19 patients)? 5. Are you concerned about inadequate protective measures? And 6. Are you concerned about the current prevention and control strategy?<br><br>For Psychological status, the GHQ-12 was employed using the 0-0-1-1 scoring method.	Out of a total of 4,600 questionnaires distributed, 4,357 were collected (94.7% response). The main concerns of healthcare workers were: infection of colleagues (72.5%), infection to family members (63.9%), protective measures (52.3%) and medical violence (48.5%). 39.1% of the healthcare workers had psychological problems, especially those who worked in Wuhan, had been on the front line, had been isolated and had infected family members or colleagues.
Zhu, et al. (2020)	Risk perception General psychological	5 questions on risk perception of COVID-19:1 Do you feel you are exposed to COVID-19? 2 Have you ever thought about giving up because of COVID-19? 3 Have	1509 of the respondents (29.8%) reported symptoms of stress, 681 (13.5%) depression, and 1218 (24.1%) anxiety. Being female (hazard ratio [HR], 1.31; P = 0.032), more than 10 years of work

	state, anxiety, insomnia, impact of the stressful event	you ever worried about dying once infected? 4 Do you feel that your family and friends have avoided contact with you because of your work? 5 Have you ever worried about you or your family members becoming infected with COVID-19?<br>5 items on psychological protection measures: (1) Have you joined the Balint Tongji WeChat group? (2) Have you received attention from hospital and department managers? (3) Are you satisfied with the full coverage of all departments of protective measures? (4) Are you satisfied with the organized work shifts? (5) Are you satisfied with the logistic support and convenience of the facilities of Tongji Hospital?<br><br>Impact of Event Scale-Revised (IES-R) para medir estrés, Patient Health Questionnaire-9 (PHQ-9) para medir depresión, and Generalized Anxiety Disorder 7-item (GAD-7) para ansiedad.	(HR, 2.02; P <0.001), concomitant chronic diseases (HR, 1.51; P <0.001), history of mental disorders (HR, 3.27; P <0.001), and having family members with the virus or suspected (HR, 1.23; P = 0.030) were risk factors for stress. Whereas, care provided by hospital and department managers (odds ratio [OR], 0.76; P = 0.024) and full coverage, with protective measures, of all departments (OR, 0.69; P = 0.004) were protective factors.
Huang, Xu, & Liu (2020)	Emotional responses and coping	Participants rated the degree to which they experienced anxiety, fear, sadness, and anger in response to the COVID-19 outbreak on a 5-point scale, ranging from 1 (no such emotion) to 5 (most intense feeling of the emotion). The order of presentation of these items was randomized.<br>Brief COPE coping questionnaire (Carver, 1997) The scale consists of 16 items divided into two types of coping: problem-focused (active coping, planning and use of instrumental support) and emotion-focused (use of emotional support, acceptance, positive	The results found that women showed more anxiety and fear. Urban participants showed more anxiety and fear than rural participants, but rural participants showed more sadness. The closer COVID-19 participants were, the more anxiety and anger. Compared to undergraduate nursing students, nurses have stronger emotional responses and use more problem-focused coping.

		reframing, religion, humor, substance use, self-harm, blaming, denial, distancing and venting behaviors).	
Jiang, et al. (2020)		The questionnaire collected self-efficacy and loneliness (five-point Likert scale: 0 (strongly disagree) to 4 (strongly agree).	There was a significant positive correlation between loneliness and self-efficacy score (β = 0.30, 95% CI = 0.12 to 0.48, p = 0.001), while a significant negative correlation was found between loneliness with age (β = -1.35, 95% CI = -2.54 to -0.17, p = 0.026), education (β = -1.41, 95% CI = -2.51 to -0.31, p = 0.012), seniority (β = -1.96, 95% CI = -3.14 to -0.78, p = 0.001) and health status (β = -5.41, 95% CI = -7.58 to -3.23, p <0.001).
Kang, et al. (2020)	Depression, anxiety, insomnia and impact of stressful event.	Patient Health Questionnaire (PHQ-9) (Kocalevent et al., 2013), as a self-report of depression severity. Categories: no depression (0-4), mild depression (5-9), moderate depression (10-14), or severe depression (15-21).<br>GAD-7 (Löwe et al., 2008). to assess the severity of anxiety. The total scores are classified as: minimal anxiety (0-4), mild anxiety (5-9), moderate anxiety (10-14), or severe anxiety (15-21).<br>ISI (Morin et al., 2011) as a measure of insomnia severity that has been shown to be valid and accountable. Total scores are classified as follows: normal (0-7), subthreshold (8-14), moderate insomnia (15-21), or severe insomnia (22-28).<br>The IES-R (Daniel and Weiss, 2007) is a self-report measure used to assess the response to a specific stressful life event. Total scores are classified as follows: subclinical (0-8), mild distress (9-25), moderate distress (26-43) and severe distress (44-88).	The results indicate that 36.9% had mental health disorders below threshold (mean PHQ-9: 2.4), 34.4% had mild disorders (mean PHQ-9: 5.4), 22.4% had moderate disorders (mean PHQ-9: 9.0), and 6.2% had severe disturbance (mean PHQ-9: 15.1).<br>The greatest burden fell on young women. Of all participants, 36.3% accessed psychological materials (such as books on mental health), 50.4% accessed psychological resources available through the media (messages, both on various on-line platforms and on television news, about coping or self-help methods), and 17.5% participated in counseling or psychotherapy. Levels of psychological distress and important factors such as exposure to infected persons and psychological assistance were identified. Although mental health resources were used sparingly, distressed staff found these services helpful in alleviating acute mental health disorders and improving their perceptions of physical health.
Lai, et al. (2020)	General psychological	Versions of the Patient Health Questionnaire (9-item PHQ), the Generalized Anxiety Disorder Scale (7-item	760 professionals (60.5%) worked in Wuhan hospitals, and 522 (41.5%) worked in front-line medical care.

	state, anxiety, insomnia, impact of the stressful event	GAD), the Insomnia Severity Index (ISI, 7-item) and the revised Impact of Events Scale (22-item IES-R),	A considerable proportion of participants reported symptoms of depression (634 [50.4%]), anxiety (560 [44.6%]), insomnia (427 [34.0%]), and stress (899 [71.5%]).<br>Nurse practitioner, women, frontline health care workers, and those working in Wuhan reported more severe ratings on all measures of mental health symptoms than other health care professionals. For example: median Patient Health Questionnaire scores for physicians vs nursing staff were 4.0 [1.0-7.0] vs 5.0 [2.0-8.0]; P - .007; median Generalized Anxiety Disorder scale scores among men vs women were 2.0 [0-6.0] vs 4.0 [1.0-7.0]; P <001; median Insomnia Severity Index scores among frontline professionals vs second-line workers was 6.0 [2.0-11.0] vs 4.0 [1.0-8.0]; P <001; median [IQR] Impact of Event Scale scores among those in Wuhan vs those in Hubei, outside Wuhan and those outside Hubei were: 21.0 [8.5-34.5] vs 18.0 [6.0- 28.0] in Hubei, outside Wuhan, and 15.0 [4.0-26.0] outside Hubei; P <001).<br>Multivariable logistic regression analyses showed that participants from outside Hubei province were associated with a lower risk of stress symptoms compared with those from Wuhan (odds ratio [OR], 0.62; 95% CI, 0.43-0.88; P=0.008).<br>Front-line health care workers involved in the direct diagnosis and treatment of patients with COVID-19 were associated with an increased risk of depressive symptoms (OR, 1.52; 95%CI, 1.11-2.09; P - .01), anxiety (OR, 1.57; 95%CI, 1.22-2.02; P <.001), insomnia (OR, 2.97; 95%CI, 1.92-4.60; P <.001), and stress (OR, 1.60; 95%CI, 1.25-2.04; P <.001).
Wu, et al. (2020)	Burnout	Maslach Burnout Inventory	The group working at the front had a lower frequency of burnout (13% vs. 39%, P < .0001), and were less worried about ending up infected compared to the usual on-call group.

Siyu, et al. (2020).	Anxiety, depression, insomnia	Zung Self-rating Anxiety Scale, Chinese version of the Center for Epidemiologic Studies Depression Scale and the Insomnia Severity Index.	Anxiety, depression, and insomnia symptoms in the population accounted for 5.9%, 28%, and 34.3% of the total sample group, respectively. Women, contact with confirmed or suspected cases, working in the front-line clinic, low total social support score and low score in each dimension, nurses and other factors are closely related to the occurrence of symptoms of depression, anxiety, insomnia. After controlling for statistically significant population characteristics variables, we found that total social support score and score on all three dimensions were protective factors for psychological problems.
